# 1-[(2-Bromo­phen­yl)di­phenyl­meth­yl]-3-(tri­fluorometh­yl)-1*H*-pyrazole–1-(tri­phenyl­meth­yl)-3-(tri­fluoro­meth­yl)-1*H*-pyrazole (0.638:0.362)

**DOI:** 10.1107/S2414314625004663

**Published:** 2025-06-17

**Authors:** Firudin I. Guseinov, Aida I. Samigullina, Tuncer Hökelek, Sahil Z. Hamidov, Jamal Lasri, Khudayar I. Hasanov, Tahir A. Javadzade, Alebel N. Belay

**Affiliations:** a Kosygin State University of Russia, 117997 Moscow, Russian Federation; b N. D. Zelinsky Institute of Organic Chemistry, Russian academy of Sciences, 119991 Moscow, Russian Federation; c Hacettepe University, Department of Physics, 06800 Beytepe-Ankara, Türkiye; d Azerbaijan Technological University, Shah Ismayil Khatai Avenue 103, AZ2011 Ganja, Azerbaijan; e Department of Chemistry Rabigh College of Science and Arts King Abdulaziz University,Jeddah 21589 Saudi Arabia; f Western Caspian University, Istiqlaliyyat Street 31, AZ 1001, Baku, Azerbaijan; g Azerbaijan Medical University, Scientific Research Centre (SCR), A. Kasumzade St. 14, AZ1096 Baku, Azerbaijan; h Department of Chemistry and Chemical Engineering, Khazar University, Mahsati St. 41, AZ1096 Baku, Azerbaijan; i Department of Chemistry, Bahir Dar University, PO Box 79, Bahir Dar, Ethiopia; University of Aberdeen, United Kingdom

**Keywords:** crystal structure, hydrogen bond, pyrazole

## Abstract

In the title compound, 0.638C_23_H_16_BrF_3_N_2_·0.362C_23_H_17_F_3_N_2_, the Br atom has been partially replaced by an H atom by reaction with NaH. In the crystal, pairwise C—H⋯Br hydrogen bonds link the mol­ecules into centrosymmetric dimers, enclosing *R*^2^_2_(16) ring motifs. A Hirshfeld surface analysis indicates that the most important contributions for the crystal packing are from H⋯H (40.1%), H⋯F/F⋯H (21.4%) and H⋯C/C⋯H (18.9%) inter­actions.

## Abstract 2

In the title disordered co-crystal, C—H⋯Br hydrogen bonds link the mol­ecules into centrosymmetric dimers, enclosing *R*^2^_2_(16) ring motifs.

## Graphical abstract

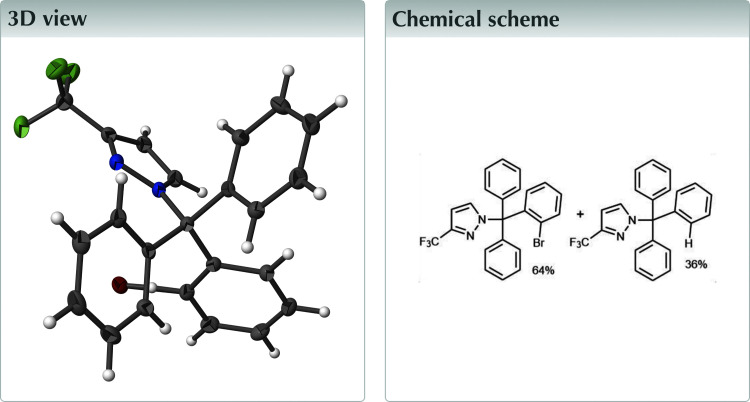

## Structure description

Among N-hetercyclic compounds, pyrazole and its derivatives constitute a versatile building block in organic synthesis and possess a wide spectrum of biological activities such as anti­fungal, anti­tubeculosis, anti­microbial and anti-inflammatory (Khalilov *et al.*, 2024 ▸). As part of our ongoing studies in this area, we report herein the synthesis and structure of the title compound, 0.638C_23_H_16_BrF_3_N_2_·0.362C_23_H_17_F_3_N_2_ (**I**), which crystallized as a co-crystal due to an inadvertent partial reaction of the [(2-bromo­phen­yl)chloro­methyl­ene]di­benzene starting material with NaH.

**Figure 1:**
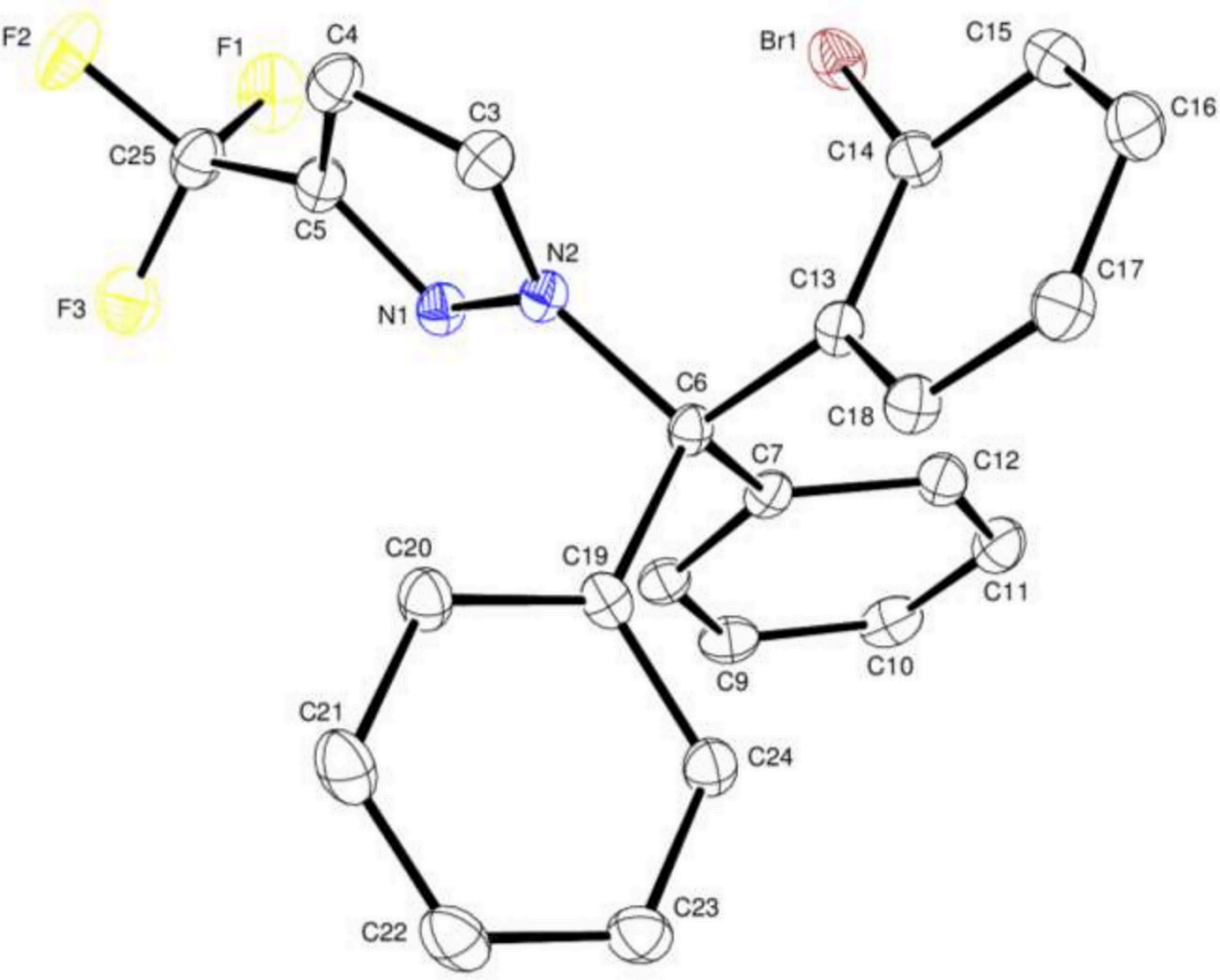
The title mol­ecule with atom-numbering scheme and displacement ellipsoids at the 50% probability level.

Compound (I) contains pyrazole *A* (N1/N2/C3–C5) and phenyl *B* (C7–C12), *C* (C13–C18) and *D* (C19–C24) rings (Fig. 1 ▸) linked at C6. They are oriented at dihedral angles of *A*/*B* = 45.31 (6)°, *A*/*C* = 70.94 (6)°, *A*/*D* = 86.87 (6)°, *B*/*C* = 72.22 (5)°, *B*/*D* = 78.29 (6)° and *C*/*D* = 74.72 (6)°. The minimum and maximum bond angles at C6 are N2—C6—C13 = 105.75 (13) and C13—C6—C19 = 112.04 (13)°, respectively. Atom C14 is bonded to bromine and hydrogen in a 0.6380 (14):0.3620 (14) ratio (see the refinement section).

**Figure 2:**
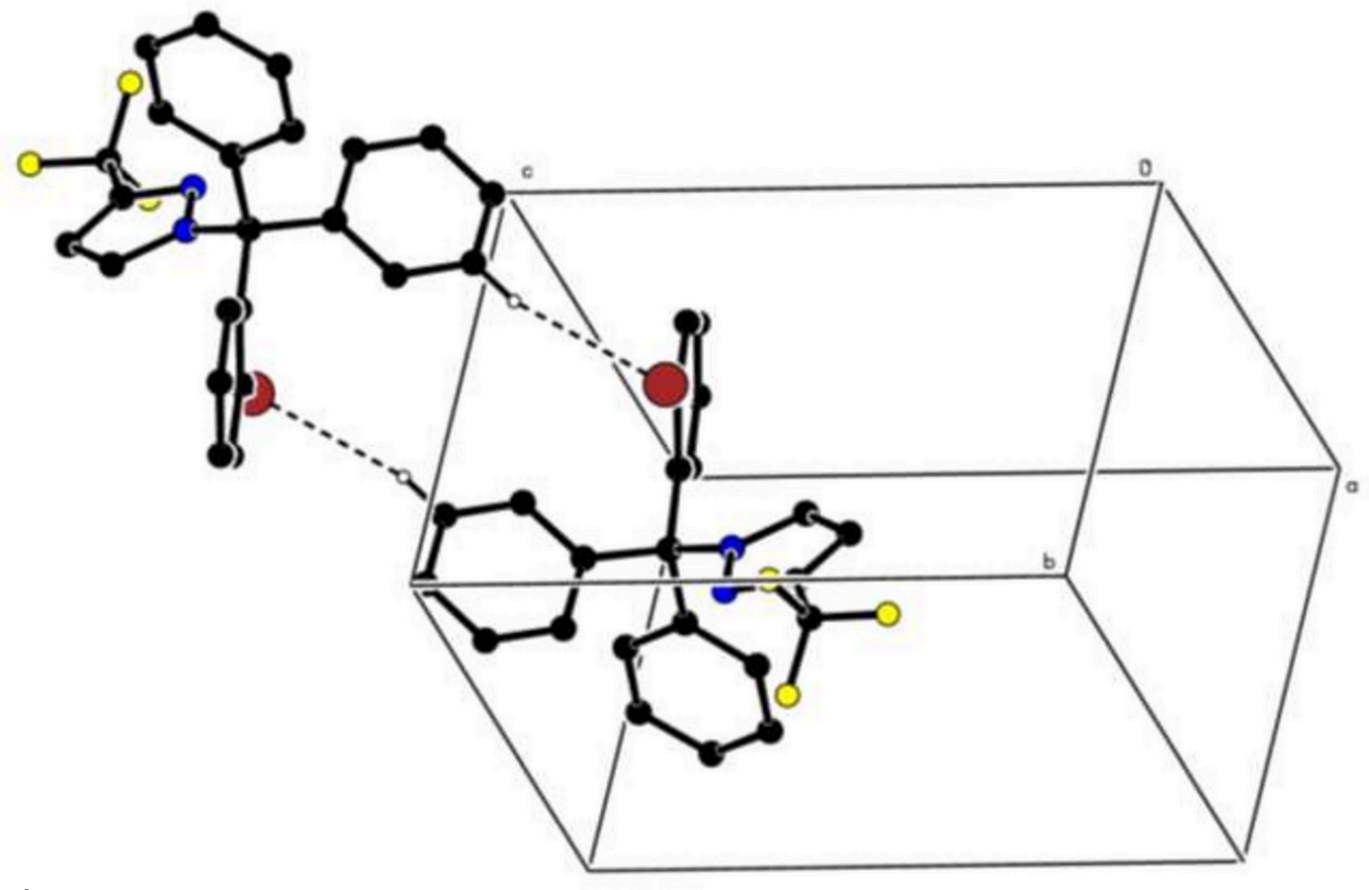
A partial packing diagram viewed approximately along the *a*-axis direction. Inter­molecular C—H⋯O hydrogen bonds are shown as dashed lines. H atoms not involved in these inter­actions have been omitted for clarity.

In the crystal, pairwise C—H⋯Br hydrogen bonds (Table 1 ▸) link the mol­ecules into centrosymmetric dimers, enclosing 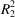
(16) ring motifs (Fig. 2 ▸). Further, there is a weak C—H⋯π inter­action (Table 1 ▸). No π–π inter­actions are observed.

**Figure 3:**
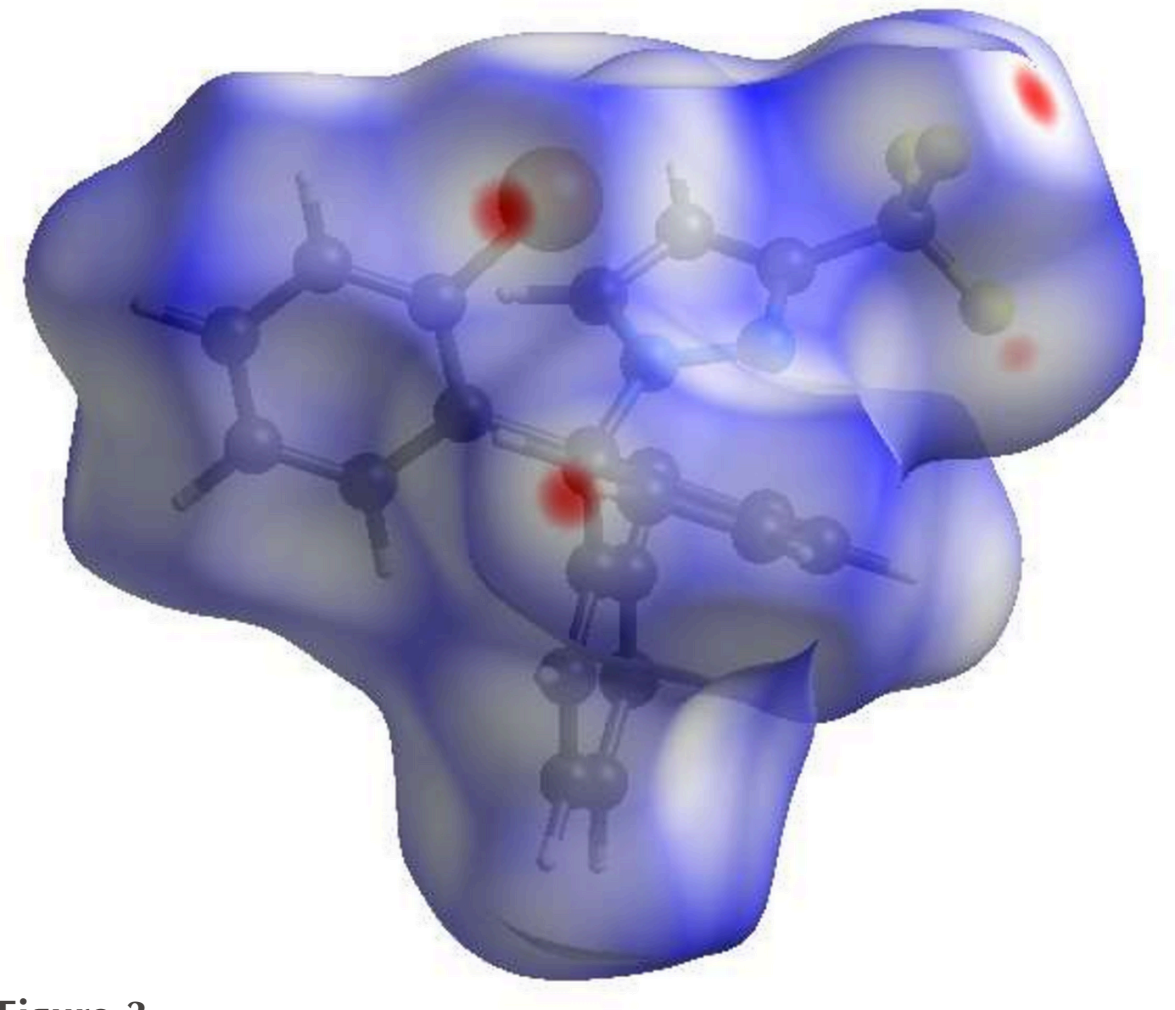
View of the three-dimensional Hirshfeld surface of the title compound plotted over *d*_norm_.

**Figure 4:**
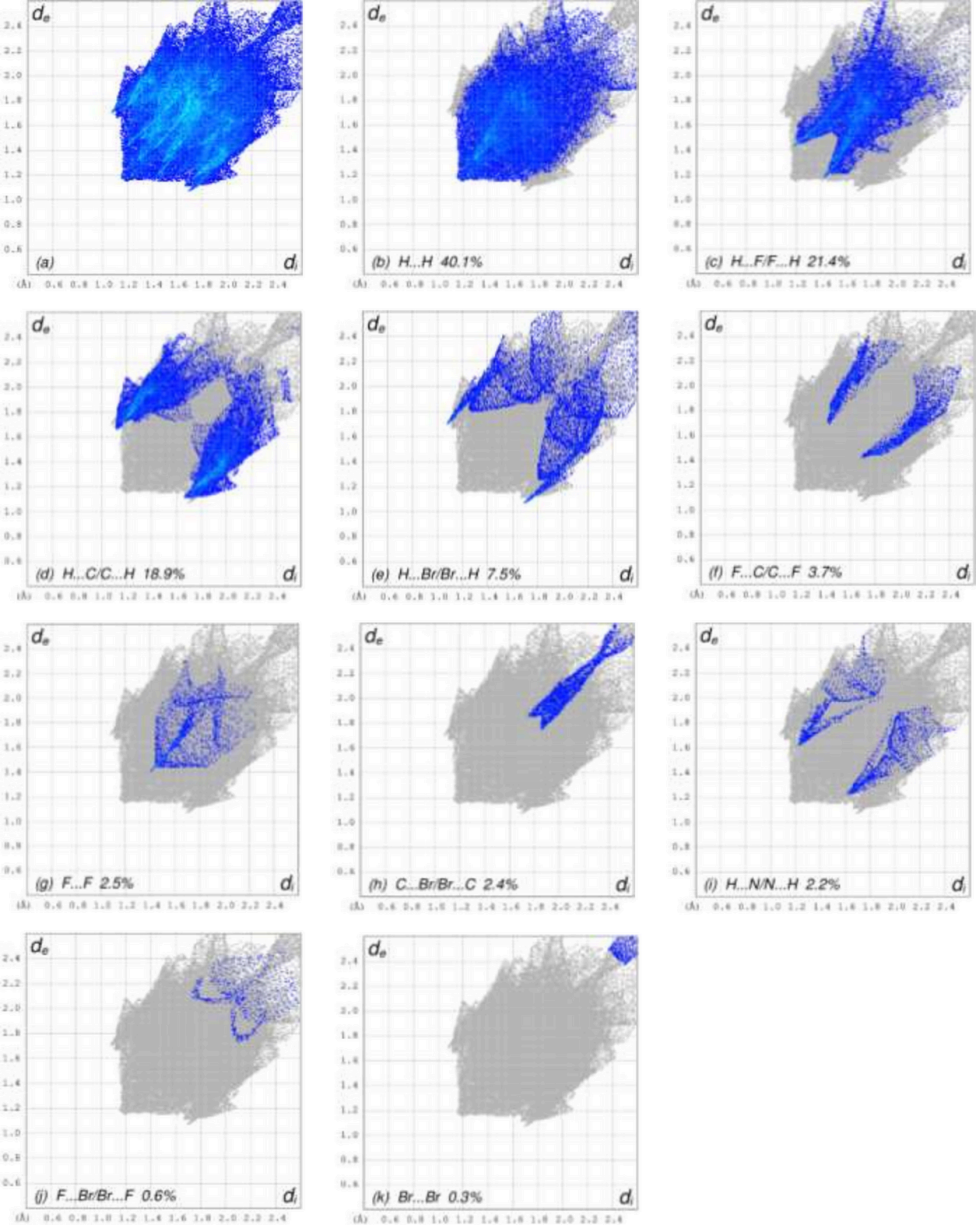
The full two-dimensional fingerprint plots for the title compound, showing (*a*) all inter­actions, and delineated into (*b*) H⋯H, (*c*) H⋯F/F⋯H, (*d*) H⋯C/C⋯H, (*e*) H⋯Br/Br⋯H, (*f*) F⋯C/C⋯F, (*g*) F⋯F, (*h*) C⋯Br/Br⋯C, (i) H⋯N/N⋯H, (*j*) F⋯Br/Br⋯F and (*k*) Br⋯Br inter­actions. The *d*_i_ and *d*_e_ values are the closest inter­nal and external distances (in Å) from given points on the Hirshfeld surface.

In order to visualize the inter­molecular inter­actions in the crystal of (I), a Hirshfeld surface analysis (Fig. 3 ▸) was carried out using *CrystalExplorer* (Spackman *et al.*, 2021 ▸). The overall two-dimensional fingerprint plot, Fig. 4 ▸*a*, and those delineated into the different contact types are illustrated in Fig. 4 ▸*b–f*, respectively.

## Synthesis and crystallization

**Figure 5:**
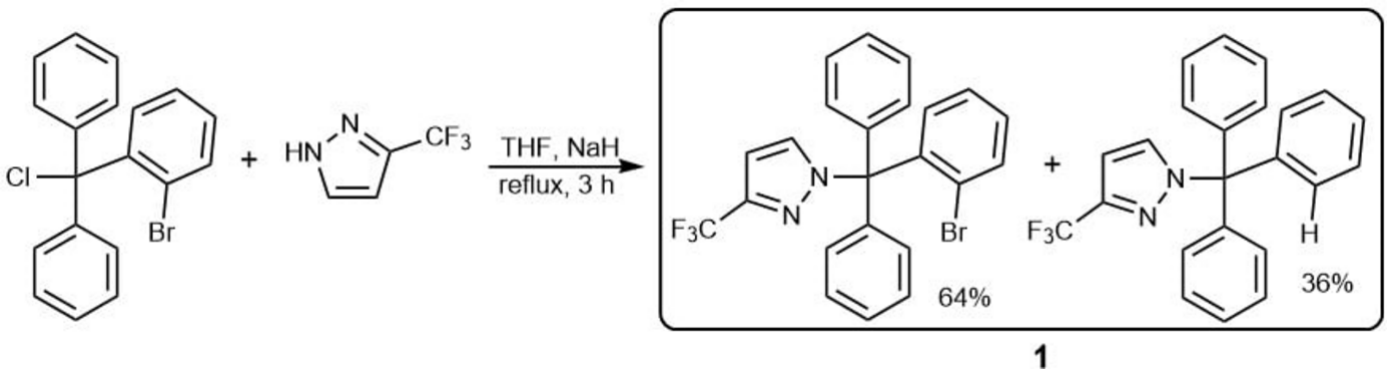
The synthesis of the title compound.

To a solution of 136 mg (1 mmol) of 3-(tri­fluoro­meth­yl)-1*H*-pyrazole in 15 ml of tetra­hydro­furan, 40 mg of 60%_wt_ NaH powder was added with stirring and the mixture was boiled for 5 min. To the resulting solution, 357 mg (1 mmol) of [(2-bromo­phen­yl)chloro­methyl­ene]di­benzene, contaminated by (chloro­methane­tri­yl)benzene formed *in situ*, in 10 ml of tetra­hydro­furan was added, and the reaction mixture was boiled for 3 h. The solvent was removed *in vacuo* and the remaining powder was recrystallized from aceto­nitrile solution. The title compound was isolated in the form of colorless prisms. yield: 375 mg (82%); m.p. 379–381 K. According to the X-ray data, the bromine atom has been partially replaced by a hydrogen atom through its reaction with the excess of sodium hydride (Rohrbach *et al.*, 2019 ▸). The refined occupancy values of atoms Br1 and H14 are 0.6380 (14) and 0.3620 (14). In fact, the elemental analysis, ^1^H NMR and ^13^C NMR data confirm the partially replacement of Br atom with the H atom in the title compound. Analysis calculated (%) for C_23_H_16.36_Br_0.64_F_3_N_2_: C 64.41, H 3.84, N 6.53; found C 60.40, H 3.82, N 6.51. ^1^H NMR (300 MHz, CDCl_3_): 6.53–7.72 (4*H*, 2CF_3_CCHCHN, 29H, 5Ph and Ar–Br). ^13^C NMR (75 MHz, CDCl_3_): 79.55, 80.35, 102.80, 103.35, 126.48, 127.00, 127.52, 128.00, 128.15, 130.07, 130.13, 130.25, 130.43, 131.38, 131.89, 132.13, 132.46, 133.61, 133.98, 135.89, 136.16, 140.47, 141.50 and 141.68. The synthesis scheme is shown in Fig. 5 ▸.

## Refinement

Crystal data, data collection and structure refinement details are summarized in Table 2 ▸. When refined with full occupancy, the bromine atom showed excessive displacement and the refinement was unstable, so the Br occupancy was allowed to vary and an H atom with the complementary occupancy factor occupying the same position bound to C14 was added to the model to treat the positional disorder. The bromine and hydrogen occupancies refined to 0.6380 (14) and 0.3620 (14), respectively. This is chemically reasonable and can be related to the presence of sodium hydride (see above).

## Supplementary Material

Crystal structure: contains datablock(s) I. DOI: 10.1107/S2414314625004663/hb4519sup1.cif

Structure factors: contains datablock(s) I. DOI: 10.1107/S2414314625004663/hb4519Isup2.hkl

Supporting information file. DOI: 10.1107/S2414314625004663/hb4519Isup3.cml

CCDC reference: 2454123

Additional supporting information:  crystallographic information; 3D view; checkCIF report

Additional supporting information:  crystallographic information; 3D view; checkCIF report

## Figures and Tables

**Table 1:** Hydrogen-bond geometry (Å, °) *Cg*2 is the centroid of the C7–C12 ring.

*D*—H⋯*A*	*D*—H	H⋯*A*	*D*⋯*A*	*D*—H⋯*A*
C11—H11⋯Br1^i^	0.95	2.90	3.8277 (19)	165
C22—H22⋯*Cg*2^ii^	0.95	2.85	3.648 (2)	143

Symmetry codes: (i) 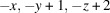; (ii) 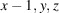.

**Table 2:** Experimental details

Crystal data	
Chemical formula	0.64(C_23_H_16_BrF_3_N_2_)·0.36(C_23_H_17_F_3_N_2_)
*M*_r_	428.88
Crystal system, space group	Triclinic, *P* 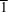
Temperature (K)	100
*a*, *b*, *c* (Å)	8.84082 (17), 9.48683 (18), 11.6797 (2)
α, β, γ (°)	95.3496 (16), 91.0661 (16), 103.5919 (17)
*V* (Å^3^)	947.18 (3)
*Z*	2
Radiation type	Cu *K*α
μ (mm^−1^)	2.42
Crystal size (mm)	0.46 × 0.25 × 0.16

Data collection	
Diffractometer	XtaLAB Synergy, Dualflex, HyPix
Absorption correction	Gaussian (*CrysAlis PRO*; Rigaku OD, 2024 ▸)
*T*_min_, *T*_max_	0.310, 1.000
No. of measured, independent and observed [*I* > 2σ(*I*)] reflections	21061, 4105, 4012
*R*_int_	0.029
(sin θ/λ)_max_ (Å^−1^)	0.640

Refinement	
*R*[*F*^2^ > 2σ(*F*^2^)], *wR*(*F*^2^), *S*	0.037, 0.095, 1.05
No. of reflections	4105
No. of parameters	263
H-atom treatment	H-atom parameters constrained
Δρ_max_, Δρ_min_ (e Å^−3^)	0.78, −0.29

Computer programs: *CrysAlis PRO* (Rigaku OD, 2024 ▸), *SHELXT2014/5* (Sheldrick, 2015*a* ▸) and *SHELXL2019/2* (Sheldrick, 2015*b* ▸).

## full crystallographic data

### 1-[(2-Bromophenyl)diphenylmethyl]-3-(trifluoromethyl)-1H-pyrazole; 1-(triphenylmethyl)-3-(trifluoromethyl)-1H-pyrazole Crystal data

**Table:**

0.64(C_23_H_16_BrF_3_N_2_)·0.36(C_23_H_17_F_3_N_2_)	*Z* = 2
*M**_r_* = 428.88	*F*(000) = 436
Triclinic, *P*1	*D*_x_ = 1.504 Mg m^−^^3^
*a* = 8.84082 (17) Å	Cu *K*α radiation, λ = 1.54184 Å
*b* = 9.48683 (18) Å	Cell parameters from 14043 reflections
*c* = 11.6797 (2) Å	θ = 3.8–80.4°
α = 95.3496 (16)°	µ = 2.42 mm^−^^1^
β = 91.0661 (16)°	*T* = 100 K
γ = 103.5919 (17)°	Prism, colorless
*V* = 947.18 (3) Å^3^	0.46 × 0.25 × 0.16 mm

### 1-[(2-Bromophenyl)diphenylmethyl]-3-(trifluoromethyl)-1H-pyrazole; 1-(triphenylmethyl)-3-(trifluoromethyl)-1H-pyrazole Data collection

**Table:**

XtaLAB Synergy, Dualflex, HyPix diffractometer	4012 reflections with *I* > 2σ(*I*)
Detector resolution: 10.0000 pixels mm^-1^	*R*_int_ = 0.029
ω scans	θ_max_ = 80.6°, θ_min_ = 3.8°
Absorption correction: gaussian (CrysAlisPro; Rigaku OD, 2024)	*h* = −8→10
*T*_min_ = 0.310, *T*_max_ = 1.000	*k* = −12→12
21061 measured reflections	*l* = −14→14
4105 independent reflections	

### 1-[(2-Bromophenyl)diphenylmethyl]-3-(trifluoromethyl)-1H-pyrazole; 1-(triphenylmethyl)-3-(trifluoromethyl)-1H-pyrazole Refinement

**Table:**

Refinement on *F*^2^	Primary atom site location: dual
Least-squares matrix: full	Hydrogen site location: inferred from neighbouring sites
*R*[*F*^2^ > 2σ(*F*^2^)] = 0.037	H-atom parameters constrained
*wR*(*F*^2^) = 0.095	*w* = 1/[σ^2^(*F*_o_^2^) + (0.0432*P*)^2^ + 0.888*P*] where *P* = (*F*_o_^2^ + 2*F*_c_^2^)/3
*S* = 1.05	(Δ/σ)_max_ < 0.001
4105 reflections	Δρ_max_ = 0.78 e Å^−^^3^
263 parameters	Δρ_min_ = −0.28 e Å^−^^3^
0 restraints	

### 1-[(2-Bromophenyl)diphenylmethyl]-3-(trifluoromethyl)-1H-pyrazole; 1-(triphenylmethyl)-3-(trifluoromethyl)-1H-pyrazole Special details

**Table:**

Geometry. All esds (except the esd in the dihedral angle between two l.s. planes) are estimated using the full covariance matrix. The cell esds are taken into account individually in the estimation of esds in distances, angles and torsion angles; correlations between esds in cell parameters are only used when they are defined by crystal symmetry. An approximate (isotropic) treatment of cell esds is used for estimating esds involving l.s. planes.

### 1-[(2-Bromophenyl)diphenylmethyl]-3-(trifluoromethyl)-1H-pyrazole; 1-(triphenylmethyl)-3-(trifluoromethyl)-1H-pyrazole Fractional atomic coordinates and isotropic or equivalent isotropic displacement parameters (Å^2^)

**Table:**

	*x*	*y*	*z*	*U*_iso_*/*U*_eq_	Occ. (<1)
Br1	0.04169 (3)	0.46569 (3)	0.70061 (3)	0.02635 (12)	0.6380 (14)
F1	0.11203 (14)	0.91657 (15)	0.49575 (12)	0.0398 (3)	
F2	0.26791 (17)	0.89368 (14)	0.36004 (10)	0.0393 (3)	
F3	0.35009 (15)	1.03658 (13)	0.51449 (11)	0.0356 (3)	
N1	0.32218 (16)	0.78802 (16)	0.63930 (12)	0.0195 (3)	
N2	0.35135 (16)	0.65782 (15)	0.65518 (12)	0.0176 (3)	
C3	0.3493 (2)	0.57369 (19)	0.55393 (15)	0.0222 (3)	
H3	0.367687	0.478646	0.544845	0.027*	
C4	0.3158 (2)	0.6514 (2)	0.46736 (15)	0.0237 (4)	
H4	0.305721	0.622763	0.386909	0.028*	
C5	0.2998 (2)	0.7824 (2)	0.52534 (15)	0.0225 (3)	
C6	0.40061 (18)	0.62246 (17)	0.76976 (14)	0.0167 (3)	
C7	0.32585 (18)	0.69732 (18)	0.86925 (14)	0.0177 (3)	
C8	0.36095 (19)	0.84936 (19)	0.88728 (15)	0.0203 (3)	
H8	0.425545	0.905119	0.835485	0.024*	
C9	0.3028 (2)	0.9205 (2)	0.97994 (16)	0.0247 (4)	
H9	0.325160	1.023968	0.989494	0.030*	
C10	0.2121 (2)	0.8406 (2)	1.05844 (16)	0.0273 (4)	
H10	0.172152	0.888721	1.121707	0.033*	
C11	0.1807 (2)	0.6893 (2)	1.04331 (16)	0.0267 (4)	
H11	0.120369	0.633808	1.097367	0.032*	
C12	0.2369 (2)	0.6185 (2)	0.94940 (15)	0.0219 (3)	
H12	0.214206	0.514998	0.939985	0.026*	
C13	0.35039 (19)	0.45490 (18)	0.76648 (14)	0.0186 (3)	
C15	0.1532 (2)	0.2253 (2)	0.73370 (16)	0.0260 (4)	
H15	0.048461	0.175161	0.713620	0.031*	
C16	0.2626 (2)	0.14738 (19)	0.75887 (16)	0.0273 (4)	
H16	0.233156	0.044104	0.756042	0.033*	
C17	0.4146 (2)	0.22167 (19)	0.78807 (16)	0.0245 (4)	
H17	0.489986	0.169482	0.805959	0.029*	
C18	0.4572 (2)	0.37302 (18)	0.79125 (14)	0.0200 (3)	
H18	0.562283	0.422246	0.810911	0.024*	
C19	0.57896 (19)	0.67975 (17)	0.78538 (15)	0.0183 (3)	
C20	0.6721 (2)	0.7239 (2)	0.69431 (16)	0.0234 (4)	
H20	0.625359	0.721401	0.620015	0.028*	
C21	0.8338 (2)	0.7717 (2)	0.71114 (18)	0.0300 (4)	
H21	0.896109	0.801274	0.648266	0.036*	
C22	0.9034 (2)	0.7763 (2)	0.81901 (18)	0.0286 (4)	
H22	1.013382	0.807270	0.830068	0.034*	
C23	0.8114 (2)	0.7352 (2)	0.91089 (17)	0.0256 (4)	
H23	0.858441	0.739789	0.985367	0.031*	
C24	0.6510 (2)	0.68749 (19)	0.89435 (15)	0.0216 (3)	
H24	0.589149	0.659670	0.957855	0.026*	
C25	0.2586 (2)	0.9072 (2)	0.47476 (16)	0.0269 (4)	
C14	0.1967 (2)	0.37605 (19)	0.73785 (15)	0.0214 (3)	
H14	0.120377	0.427587	0.720820	0.026*	0.3620 (14)

### 1-[(2-Bromophenyl)diphenylmethyl]-3-(trifluoromethyl)-1H-pyrazole; 1-(triphenylmethyl)-3-(trifluoromethyl)-1H-pyrazole Atomic displacement parameters (Å^2^)

**Table:**

	*U*^11^	*U*^22^	*U*^33^	*U*^12^	*U*^13^	*U*^23^
Br1	0.01588 (16)	0.02709 (17)	0.03626 (19)	0.00369 (11)	0.00152 (11)	0.00758 (12)
F1	0.0296 (6)	0.0501 (8)	0.0494 (8)	0.0226 (5)	0.0046 (5)	0.0198 (6)
F2	0.0574 (8)	0.0450 (7)	0.0225 (6)	0.0226 (6)	0.0014 (5)	0.0120 (5)
F3	0.0403 (7)	0.0283 (6)	0.0396 (7)	0.0086 (5)	−0.0028 (5)	0.0107 (5)
N1	0.0179 (7)	0.0209 (7)	0.0218 (7)	0.0074 (5)	0.0010 (5)	0.0055 (5)
N2	0.0170 (6)	0.0198 (7)	0.0178 (7)	0.0074 (5)	0.0012 (5)	0.0037 (5)
C3	0.0228 (8)	0.0246 (8)	0.0210 (8)	0.0094 (6)	0.0028 (6)	0.0014 (6)
C4	0.0224 (8)	0.0312 (9)	0.0196 (8)	0.0098 (7)	0.0025 (6)	0.0041 (7)
C5	0.0183 (8)	0.0296 (9)	0.0217 (8)	0.0077 (7)	0.0020 (6)	0.0072 (7)
C6	0.0159 (7)	0.0185 (7)	0.0175 (7)	0.0063 (6)	0.0018 (6)	0.0050 (6)
C7	0.0132 (7)	0.0246 (8)	0.0173 (7)	0.0077 (6)	0.0008 (6)	0.0041 (6)
C8	0.0183 (8)	0.0231 (8)	0.0209 (8)	0.0075 (6)	−0.0021 (6)	0.0026 (6)
C9	0.0230 (8)	0.0266 (9)	0.0253 (9)	0.0097 (7)	−0.0034 (7)	−0.0025 (7)
C10	0.0225 (9)	0.0404 (10)	0.0214 (8)	0.0144 (8)	0.0003 (7)	−0.0021 (7)
C11	0.0197 (8)	0.0392 (10)	0.0222 (8)	0.0073 (7)	0.0049 (7)	0.0070 (7)
C12	0.0175 (8)	0.0261 (8)	0.0230 (8)	0.0057 (6)	0.0020 (6)	0.0060 (7)
C13	0.0188 (8)	0.0196 (8)	0.0183 (7)	0.0055 (6)	0.0023 (6)	0.0044 (6)
C15	0.0242 (9)	0.0231 (9)	0.0279 (9)	0.0004 (7)	−0.0020 (7)	0.0030 (7)
C16	0.0345 (10)	0.0181 (8)	0.0290 (9)	0.0048 (7)	−0.0002 (8)	0.0051 (7)
C17	0.0268 (9)	0.0223 (8)	0.0272 (9)	0.0106 (7)	0.0012 (7)	0.0045 (7)
C18	0.0192 (8)	0.0211 (8)	0.0212 (8)	0.0072 (6)	0.0014 (6)	0.0035 (6)
C19	0.0154 (7)	0.0160 (7)	0.0251 (8)	0.0058 (6)	0.0032 (6)	0.0037 (6)
C20	0.0201 (8)	0.0265 (8)	0.0251 (9)	0.0064 (7)	0.0041 (7)	0.0071 (7)
C21	0.0201 (9)	0.0334 (10)	0.0372 (10)	0.0051 (7)	0.0097 (7)	0.0089 (8)
C22	0.0158 (8)	0.0286 (9)	0.0423 (11)	0.0055 (7)	0.0008 (7)	0.0074 (8)
C23	0.0200 (8)	0.0264 (9)	0.0311 (9)	0.0063 (7)	−0.0037 (7)	0.0045 (7)
C24	0.0198 (8)	0.0226 (8)	0.0238 (8)	0.0065 (6)	0.0017 (6)	0.0056 (6)
C25	0.0269 (9)	0.0329 (10)	0.0241 (9)	0.0114 (7)	0.0010 (7)	0.0081 (7)
C14	0.0185 (8)	0.0224 (8)	0.0242 (8)	0.0057 (6)	−0.0003 (6)	0.0045 (6)

### 1-[(2-Bromophenyl)diphenylmethyl]-3-(trifluoromethyl)-1H-pyrazole; 1-(triphenylmethyl)-3-(trifluoromethyl)-1H-pyrazole Geometric parameters (Å, º)

**Table:**

Br1—C14	1.8400 (17)	C11—H11	0.9500
F1—C25	1.345 (2)	C12—H12	0.9500
F2—C25	1.340 (2)	C13—C18	1.398 (2)
F3—C25	1.338 (2)	C13—C14	1.406 (2)
N1—C5	1.336 (2)	C15—C14	1.387 (2)
N1—N2	1.3481 (19)	C15—C16	1.391 (3)
N2—C3	1.361 (2)	C15—H15	0.9500
N2—C6	1.491 (2)	C16—C17	1.383 (3)
C3—C4	1.372 (2)	C16—H16	0.9500
C3—H3	0.9500	C17—C18	1.393 (2)
C4—C5	1.397 (3)	C17—H17	0.9500
C4—H4	0.9500	C18—H18	0.9500
C5—C25	1.487 (2)	C19—C20	1.392 (2)
C6—C13	1.543 (2)	C19—C24	1.400 (2)
C6—C19	1.544 (2)	C20—C21	1.399 (3)
C6—C7	1.545 (2)	C20—H20	0.9500
C7—C12	1.391 (2)	C21—C22	1.384 (3)
C7—C8	1.397 (2)	C21—H21	0.9500
C8—C9	1.392 (2)	C22—C23	1.388 (3)
C8—H8	0.9500	C22—H22	0.9500
C9—C10	1.389 (3)	C23—C24	1.388 (2)
C9—H9	0.9500	C23—H23	0.9500
C10—C11	1.390 (3)	C24—H24	0.9500
C10—H10	0.9500	C14—H14	0.9500
C11—C12	1.393 (3)		
			
C5—N1—N2	103.84 (14)	C14—C15—H15	119.9
N1—N2—C3	112.03 (14)	C16—C15—H15	119.9
N1—N2—C6	122.28 (13)	C17—C16—C15	119.36 (17)
C3—N2—C6	125.20 (14)	C17—C16—H16	120.3
N2—C3—C4	107.44 (15)	C15—C16—H16	120.3
N2—C3—H3	126.3	C16—C17—C18	120.02 (16)
C4—C3—H3	126.3	C16—C17—H17	120.0
C3—C4—C5	103.74 (16)	C18—C17—H17	120.0
C3—C4—H4	128.1	C17—C18—C13	122.20 (16)
C5—C4—H4	128.1	C17—C18—H18	118.9
N1—C5—C4	112.94 (16)	C13—C18—H18	118.9
N1—C5—C25	119.50 (16)	C20—C19—C24	118.32 (16)
C4—C5—C25	127.54 (16)	C20—C19—C6	122.24 (15)
N2—C6—C13	105.75 (13)	C24—C19—C6	119.44 (15)
N2—C6—C19	108.08 (13)	C19—C20—C21	120.63 (17)
C13—C6—C19	112.04 (13)	C19—C20—H20	119.7
N2—C6—C7	111.89 (12)	C21—C20—H20	119.7
C13—C6—C7	111.37 (13)	C22—C21—C20	120.31 (17)
C19—C6—C7	107.72 (13)	C22—C21—H21	119.8
C12—C7—C8	118.19 (15)	C20—C21—H21	119.8
C12—C7—C6	122.00 (15)	C21—C22—C23	119.54 (17)
C8—C7—C6	119.47 (14)	C21—C22—H22	120.2
C9—C8—C7	121.07 (16)	C23—C22—H22	120.2
C9—C8—H8	119.5	C24—C23—C22	120.24 (17)
C7—C8—H8	119.5	C24—C23—H23	119.9
C10—C9—C8	120.16 (17)	C22—C23—H23	119.9
C10—C9—H9	119.9	C23—C24—C19	120.94 (16)
C8—C9—H9	119.9	C23—C24—H24	119.5
C9—C10—C11	119.21 (17)	C19—C24—H24	119.5
C9—C10—H10	120.4	F3—C25—F2	107.32 (15)
C11—C10—H10	120.4	F3—C25—F1	105.90 (16)
C10—C11—C12	120.46 (17)	F2—C25—F1	106.31 (15)
C10—C11—H11	119.8	F3—C25—C5	113.50 (15)
C12—C11—H11	119.8	F2—C25—C5	110.83 (16)
C7—C12—C11	120.86 (17)	F1—C25—C5	112.53 (15)
C7—C12—H12	119.6	C15—C14—C13	122.00 (16)
C11—C12—H12	119.6	C15—C14—Br1	115.78 (13)
C18—C13—C14	116.31 (15)	C13—C14—Br1	122.21 (13)
C18—C13—C6	121.25 (15)	C15—C14—H14	119.0
C14—C13—C6	122.44 (14)	C13—C14—H14	119.0
C14—C15—C16	120.11 (17)	Br1—C14—H14	3.4
			
C5—N1—N2—C3	0.76 (18)	C19—C6—C13—C14	−171.36 (15)
C5—N1—N2—C6	173.14 (14)	C7—C6—C13—C14	67.9 (2)
N1—N2—C3—C4	−0.6 (2)	C14—C15—C16—C17	0.1 (3)
C6—N2—C3—C4	−172.71 (15)	C15—C16—C17—C18	−0.5 (3)
N2—C3—C4—C5	0.17 (19)	C16—C17—C18—C13	0.5 (3)
N2—N1—C5—C4	−0.65 (19)	C14—C13—C18—C17	−0.1 (3)
N2—N1—C5—C25	177.85 (15)	C6—C13—C18—C17	−179.57 (15)
C3—C4—C5—N1	0.3 (2)	N2—C6—C19—C20	−13.3 (2)
C3—C4—C5—C25	−178.05 (17)	C13—C6—C19—C20	102.79 (18)
N1—N2—C6—C13	154.86 (14)	C7—C6—C19—C20	−134.39 (16)
C3—N2—C6—C13	−33.8 (2)	N2—C6—C19—C24	166.99 (14)
N1—N2—C6—C19	−85.00 (17)	C13—C6—C19—C24	−76.89 (18)
C3—N2—C6—C19	86.35 (18)	C7—C6—C19—C24	45.93 (19)
N1—N2—C6—C7	33.4 (2)	C24—C19—C20—C21	1.3 (3)
C3—N2—C6—C7	−155.22 (15)	C6—C19—C20—C21	−178.38 (16)
N2—C6—C7—C12	123.47 (16)	C19—C20—C21—C22	−0.2 (3)
C13—C6—C7—C12	5.3 (2)	C20—C21—C22—C23	−1.0 (3)
C19—C6—C7—C12	−117.88 (16)	C21—C22—C23—C24	1.1 (3)
N2—C6—C7—C8	−63.35 (19)	C22—C23—C24—C19	0.0 (3)
C13—C6—C7—C8	178.53 (14)	C20—C19—C24—C23	−1.2 (3)
C19—C6—C7—C8	55.31 (18)	C6—C19—C24—C23	178.47 (15)
C12—C7—C8—C9	−2.9 (2)	N1—C5—C25—F3	48.2 (2)
C6—C7—C8—C9	−176.34 (15)	C4—C5—C25—F3	−133.53 (19)
C7—C8—C9—C10	2.0 (3)	N1—C5—C25—F2	169.06 (16)
C8—C9—C10—C11	0.1 (3)	C4—C5—C25—F2	−12.7 (3)
C9—C10—C11—C12	−1.2 (3)	N1—C5—C25—F1	−72.0 (2)
C8—C7—C12—C11	1.8 (2)	C4—C5—C25—F1	106.2 (2)
C6—C7—C12—C11	175.07 (15)	C16—C15—C14—C13	0.4 (3)
C10—C11—C12—C7	0.2 (3)	C16—C15—C14—Br1	179.13 (15)
N2—C6—C13—C18	125.63 (16)	C18—C13—C14—C15	−0.3 (3)
C19—C6—C13—C18	8.1 (2)	C6—C13—C14—C15	179.15 (16)
C7—C6—C13—C18	−112.61 (17)	C18—C13—C14—Br1	−179.04 (13)
N2—C6—C13—C14	−53.84 (19)	C6—C13—C14—Br1	0.5 (2)

### 1-[(2-Bromophenyl)diphenylmethyl]-3-(trifluoromethyl)-1H-pyrazole; 1-(triphenylmethyl)-3-(trifluoromethyl)-1H-pyrazole Hydrogen-bond geometry (Å, º)

*Cg*2 is the centroid of the C7–C12 ring.

**Table:**

*D*—H···*A*	*D*—H	H···*A*	*D*···*A*	*D*—H···*A*
C11—H11···Br1^i^	0.95	2.90	3.8277 (19)	165
C22—H22···*Cg*2^ii^	0.95	2.85	3.648 (2)	143

Symmetry codes: (i) −*x*, −*y*+1, −*z*+2; (ii) *x*−1, *y*, *z*.
